# Cutaneous Sebaceous Carcinoma Presenting as a Large Fungating Breast Tumour in Synchronicity With Primary Carcinomata of the Breasts

**DOI:** 10.7759/cureus.28896

**Published:** 2022-09-07

**Authors:** Stanislau Makaranka, Mikaela Frixou, Ahmed Mustafa, Ehab Husain

**Affiliations:** 1 Plastic Surgery, Aberdeen Royal Infirmary, Aberdeen, GBR; 2 Breast Surgery, Aberdeen Royal Infirmary, Aberdeen, GBR; 3 Pathology, Aberdeen Royal Infirmary, Aberdeen, GBR

**Keywords:** fungating breast mass, synchronous breast cancer, nonmelanoma skin cancer, muir-torre syndrome, extraocular sebaceous carcinoma

## Abstract

Sebaceous carcinomas are rare malignant tumours which arise from sebaceous glands. They are subclassified into ocular and extraocular subtypes and most commonly occur in the head and neck region. Tumours below the neck occur infrequently, and most commonly resemble benign skin lesions such as pyogenic granulomata and molluscum contagiosum, or malignant skin tumours like basal and squamous cell carcinomas (SCCs). We report a case of an 86-year-old lady presenting with a fungating breast tumour which began as a “mole” and exhibited insidious growth over five years to reach a maximum size of 10 cm. An excision biopsy was performed by the breast surgery team and histopathological analysis revealed a sebaceous carcinoma arising from the skin adnexa. On subsequent follow up, the patient was found to have a 19 mm mass in the left breast and a 20 mm mass in the right breast, which was P5 and P3 on clinical palpation, respectively. Core biopsies of left and right breast lesions showed invasive lobular carcinoma and invasive ductal carcinoma with lobular features respectively; the patient was started on primary letrozole treatment. The patient also went on to have a 2 cm wide local excision of the sebaceous carcinoma scar which was excised down to the pectoralis fascia. This is a unique presentation of a sebaceous gland carcinoma presenting as a fungating breast tumour. These tumours have a high metastatic potential and local recurrence rate, and can co-exist with primary carcinoma of the breast.

## Introduction

Sebaceous carcinomas are rare malignant tumours which arise from sebaceous glands. They are sub-classified into ocular and extraocular subtypes [1].

Between the years 2000 and 2016, the incidence of sebaceous carcinoma in the United States was 2.4 cases per 1 million persons annually, with a yearly average of 800 cases [2]. It is an aggressive skin cancer with a five-year mortality rate of 20% and risk factors include increasing age, male sex, Muir-Torre syndrome, and immunosuppression [2]. The incidence in males is 3.5 cases per 1 million persons compared to 1.7 cases per 1 million persons in females, with the majority of cases observed in those over 80 years of age [2]. Incidence amongst the white population is nearly three times that of non-whites, suggesting that ultraviolet radiation may contribute to the development of sebaceous carcinoma, and factors including black race, male sex, and extraocular location are associated with increased mortality [3]. The anatomical distribution of sebaceous carcinoma correlates to the distribution of sebaceous glands in the body as well as areas more prone to sun exposure; 73% of cutaneous sebaceous carcinomas present in the head and neck region [2,4].

Ocular cutaneous sebaceous carcinomas commonly present as pink or yellow nodules, with upper eyelid involvement more common than lower eyelid [4]. Extraocular tumours often present as nodules or plaques which can be associated with ulceration [5,6]. They can resemble benign skin lesions such as pyogenic granulomata and molluscum contagiosum, or malignant skin tumours like basal cell carcinomas (BCCs) and squamous cell carcinomas (SCCs) [7]. Here, we report a case of a cutaneous sebaceous carcinoma presenting as a large fungating breast tumour, associated with synchronous malignancies arising from the breast parenchyma.

## Case presentation

The patient was an 86-year-old lady who presented to a one-stop breast clinic with a large fungating mass arising from the upper inner quadrant of the left breast. The patient described the mass first appearing as a mole five years prior to presentation, and steadily increasing in size over the last two years. She had a past medical history of hypertension, rheumatoid arthritis, and bilateral hip replacements. She had no family history of breast or other cancers. Her regular medications included atorvastatin, lisinopril, and verapamil. The last mammogram was performed 15 years prior to this presentation, and it was reported as normal.

**Figure 1:**
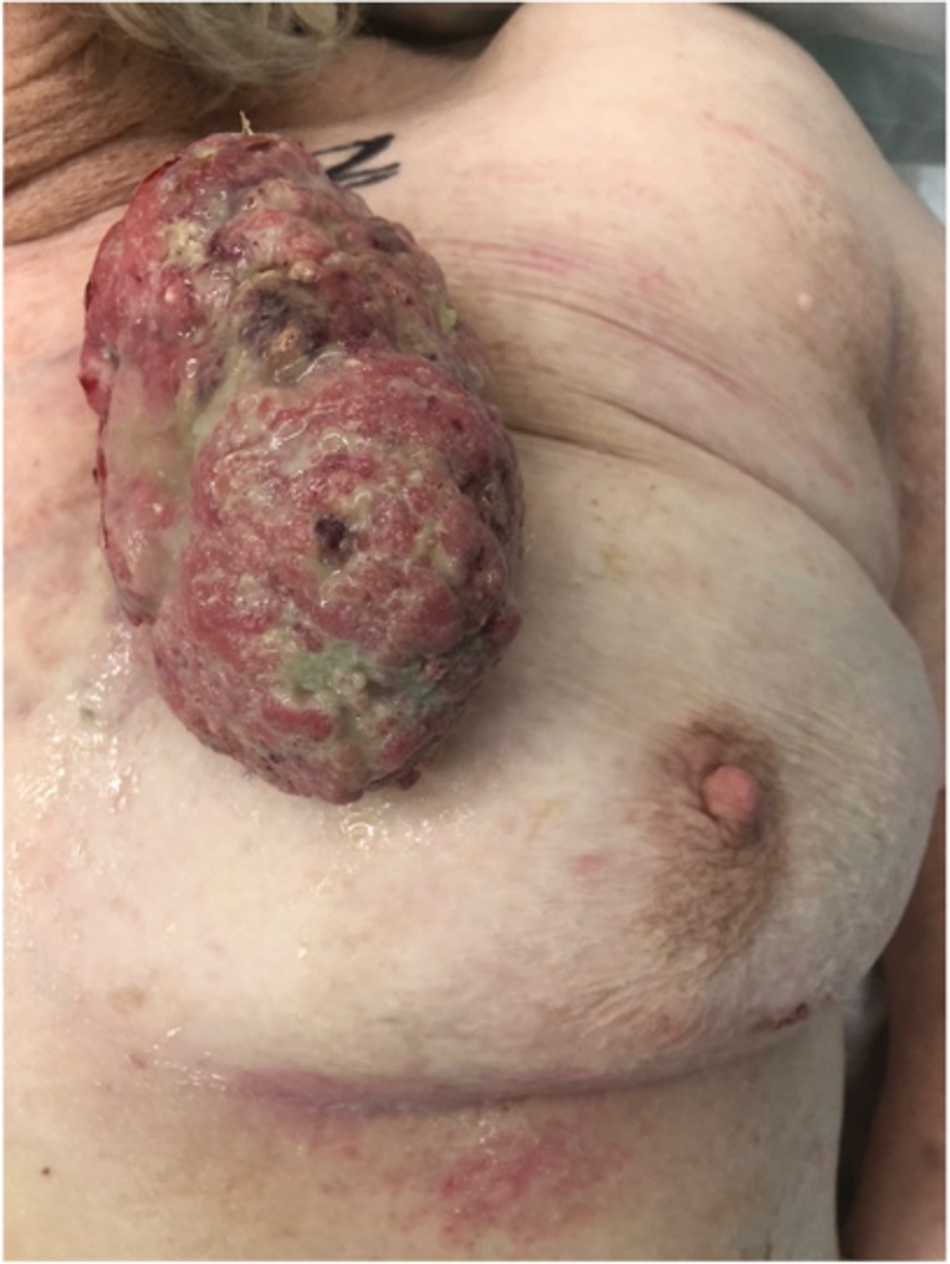
10 x 8 cm pedunculated fungating mass, arising from the upper inner quadrant of the left breast

On examination, the patient had a normal right breast and axilla (P1). The left breast had a large 10 x 8 cm pedunculated fungating mass arising from the upper inner quadrant; the remaining breast tissue was unremarkable, however, there was a palpable soft lymph node in the left axilla, which was considered suspicious (P4) (Figure 1). The mass was mobile and was not tethered to the underlying breast tissue. However, the large size of the mass precluded a thorough examination and investigations in the form of mammography and ultrasound being performed during the appointment. Following a discussion with the patient and her daughter, it was agreed that the lady would undergo an excision biopsy of the mass in the first instance, following which a thorough examination and imaging investigations would be performed.

**Figure 2:**
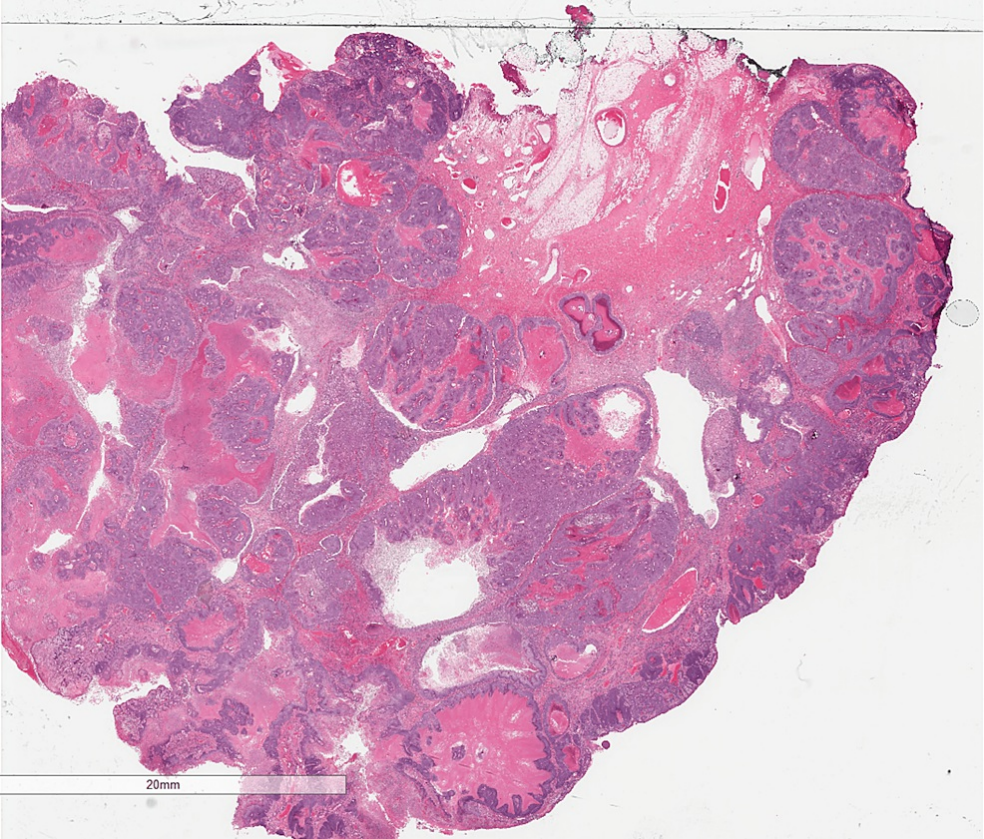
Haematoxylin and eosin (H&E) stain showing a section of the lesion

**Figure 3:**
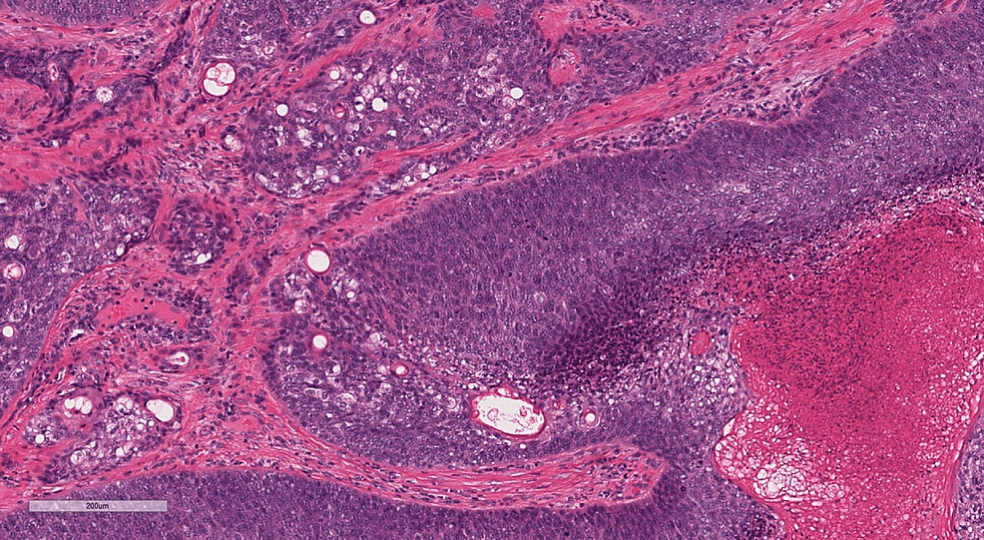
Haematoxylin and eosin (H&E) stain of the lesion showing areas of sebaceous differentiation and necrosis

**Figure 4:**
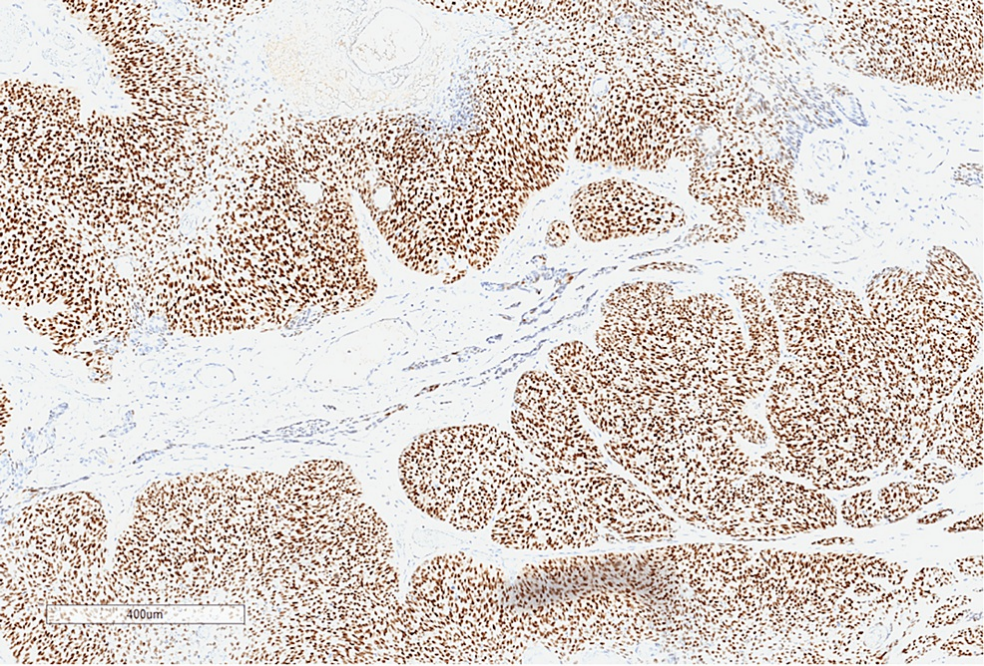
Immunohistochemistry highlighting androgen receptor positivity in the sebaceous component

**Figure 5:**
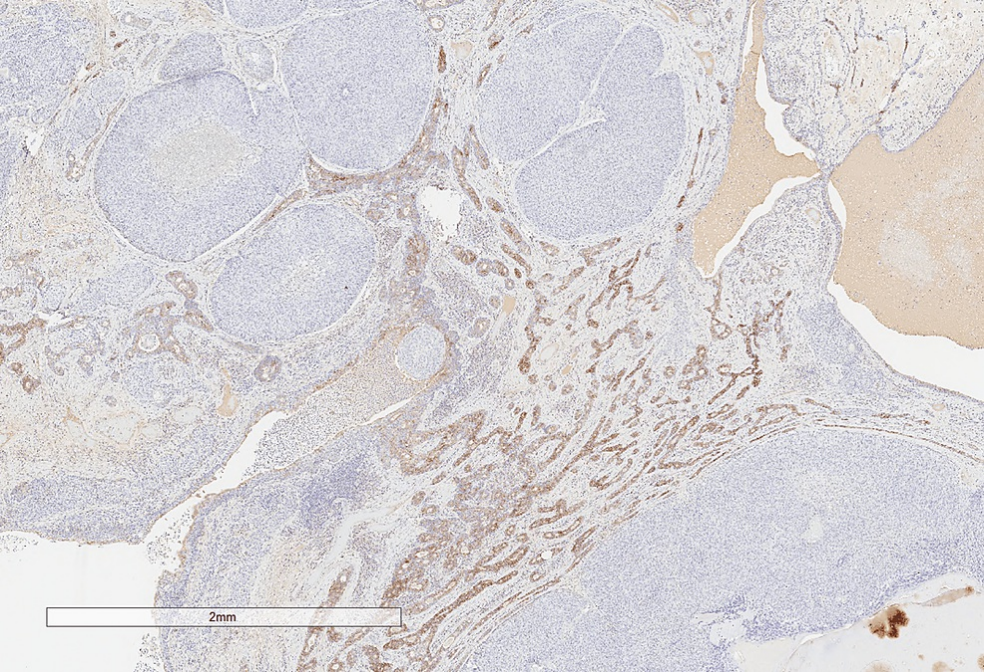
Immunohistochemistry showing that BER-Ep4 is negative in the sebaceous component, but is positive in the sweat gland component

**Figure 6:**
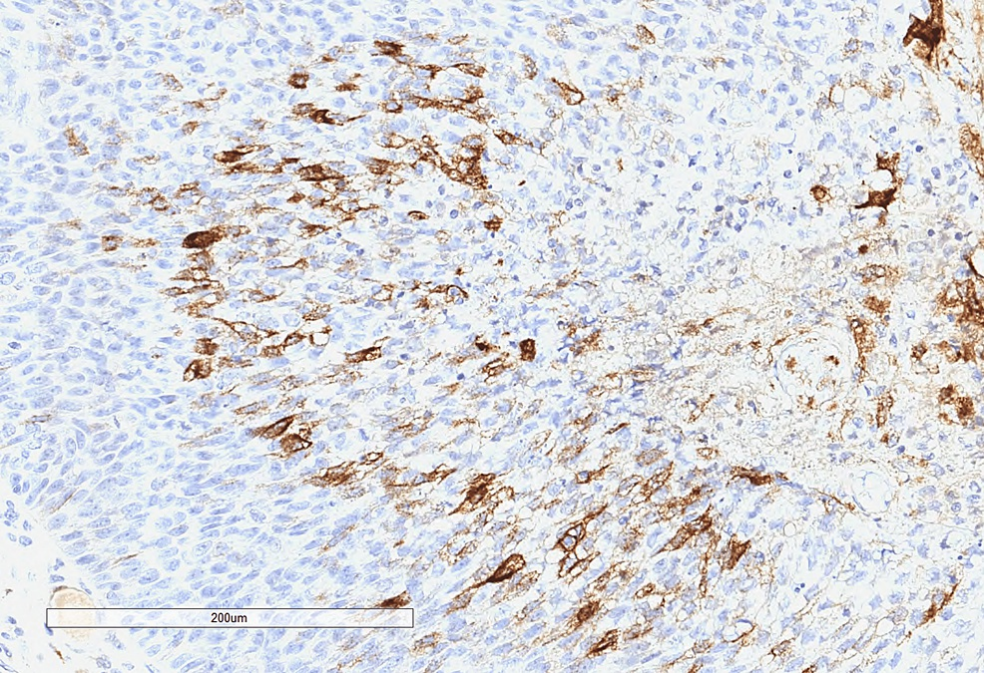
Immunohistochemistry showing epithelial membrane antigen (EMA) positivity in the sebaceous component

The patient underwent an excision biopsy of the lesion by the breast team under local anaesthesia as a day case procedure; the excised specimen was sent to the lab for histopathological analysis. On the haematoxylin and eosin (H&E) stained sections, the tumour was extensively ulcerated and originated from the skin, rather than from the breast parenchyma (Figure 2). It comprised basaloid islands of atypical cells with areas of necrosis, high mitotic activity and numerous apoptotic bodies. Focally, there were clusters of cells with pale vesicular cytoplasm in keeping with sebaceous differentiation (Figure 3). No peripheral palisading of cells was seen and surrounding artificial clefts were not identified. In addition to the sebaceous element, infiltrating acinar structures containing necrotic debris and neutrophils surrounded by a fibrous stroma were identified. These acinar structures exhibited a different pattern of immunostaining, being BER-Ep4 positive, and they were considered a sweat gland component. An extensive panel of immunohistochemistry was performed and the sebaceous component was positive for androgen receptor, p63 and GATA-3, with focal positivity for epithelial membrane antigen (EMA), but negative for BER-Ep4 (Figures 4-6). CAM 5.2 and cytokeratin-7 highlighted the acinar areas. A histopathological diagnosis of a malignant cutaneous tumour with predominant sebaceous differentiation (sebaceous carcinoma) and associated areas of sweat gland differentiation was given.

The tumour was incompletely excised at the deep margin. Following the discussion at the breast multidisciplinary meeting (MDT), the patient was referred to the plastic surgery team and the skin MDT. Recommendations from the skin MDT included a staging CT scan, ultrasound-guided fine needle aspiration (FNA) of the palpable node in the left axilla, and a 2 cm wide-local excision (WLE) of the sebaceous carcinoma scar, with consideration of adjuvant radiotherapy.

**Figure 7:**
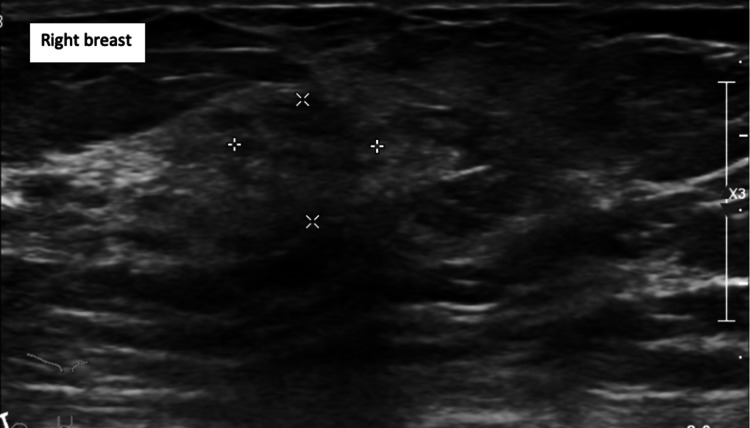
Ultrasonography of the right breast showing U4 lesion

**Figure 8:**
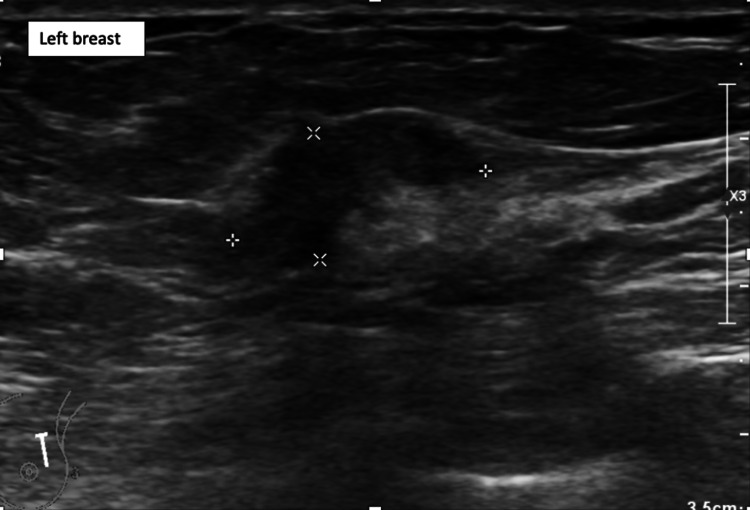
Ultrasonography of the left breast showing U5 lesion

On subsequent follow-up at the breast clinic, three months after the excision biopsy, clinical examination revealed a 20 mm mass at the 12 o’clock position of the left breast (P5), which was separate from the sebaceous carcinoma scar, and a 20 mm lesion palpable in the 9 o’clock position of the right breast (P3). Mammography of the left breast revealed a 19 mm spiculated mass at the 12 o’clock position (M5) and the right side showed a 20 mm area of distortion at the 9 o’clock position (M3). Ultrasonography of the right and left breasts and axillae showed U4, N2 and U5, N3 lesions, respectively (Figures 7-8). Ultrasound-guided FNA of the left axillary lymph node showed a highly cellular aspirate, comprising a population of lymphocytes with histiocytes and occasional tingible body macrophages, in keeping with a reactive node (LC2). Core biopsies of both breast lesions were performed.

Histopathological analysis of the right breast lesion showed a grade 2 invasive breast carcinoma of no special type with lobular features; oestrogen receptor (ER) score was 8, progesterone receptor (PR) score was 4, and human epidermal growth factor receptor-2 (HER-2) was negative. The biopsy from the left breast lesion showed a grade 2 invasive classical lobular carcinoma with classical lobular carcinoma in situ, ER8, PR5, and negative HER-2.

**Figure 9:**
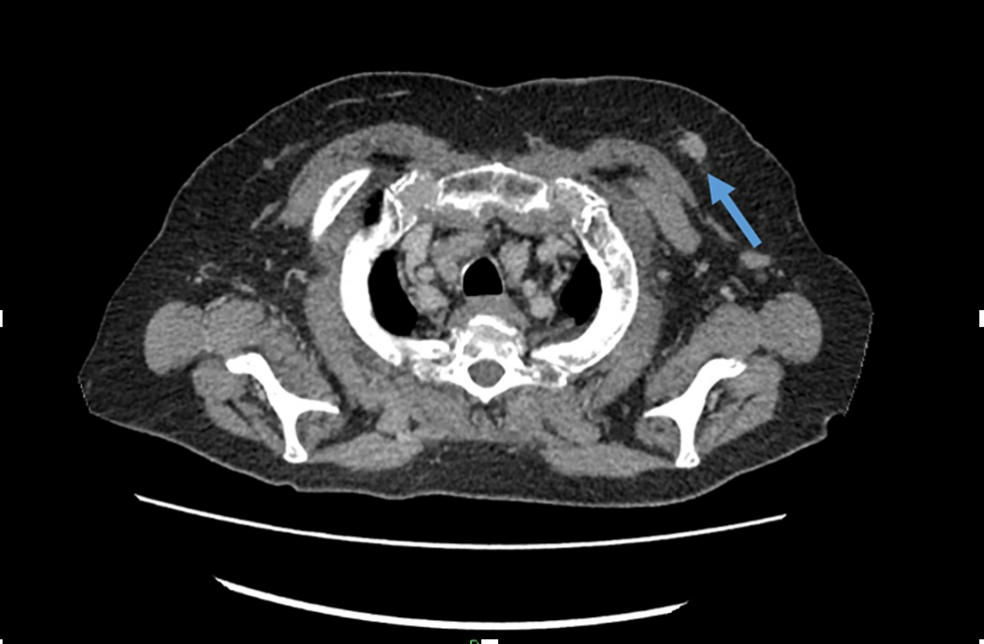
CT scan showing left upper quadrant breast lesion (arrow)

**Figure 10:**
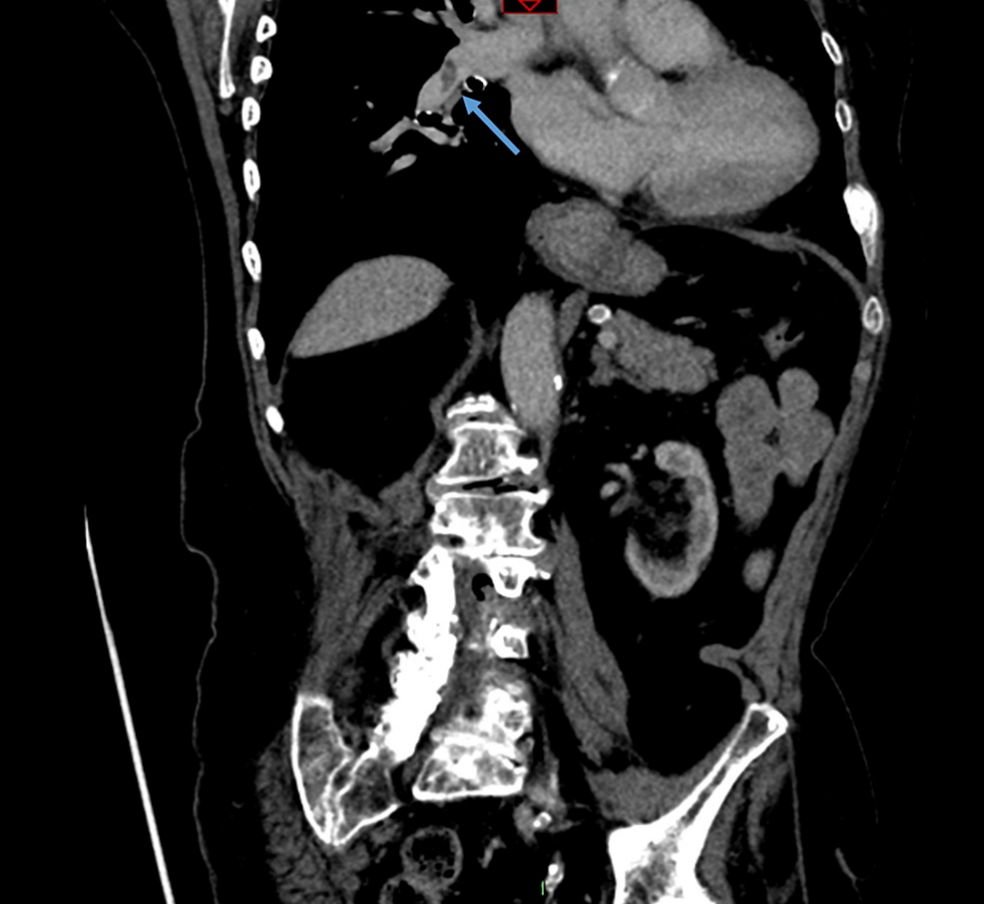
CT scan showing small pulmonary embolus (arrow)

Staging CT thorax, abdomen, and pelvis was performed and did not show any evidence of metastatic disease. It did, however, show a left upper quadrant spiculated breast lesion (which was already biopsied) and an incidental finding of bilateral small volume pulmonary emboli (PE) (Figures 9-10). The patient was started on rivaroxaban treatment for the PEs with a planned duration of treatment of six months. 

Post follow-up by the breast team with biopsy results, and discussion with the patient and her daughter, a joint decision was made to start primary letrozole treatment for both oestrogen-receptor positive breast cancers. The patient did not wish to consider surgical excision of the breast tumours, and this was thought to be a reasonable decision given the patient’s other health problems and general frailty. She did however agree to have a 2 cm WLE of the sebaceous carcinoma scar, which was excised down to pectoralis fascia by the plastic surgery team as a day case procedure, five months after initial presentation to the one stop breast clinic. There were no postoperative complications and the patient was discharged home on the same day after the procedure. Histopathological analysis of the WLE specimen showed no residual sebaceous carcinoma.

The patient was reviewed at the breast clinic three months following the initiation of letrozole treatment, where it was noted that the left breast lesion had clinically decreased in size, and the right breast lesion had softened, indicating a good response to treatment. She reported no side effects from the letrozole treatment. The patient is due to be followed up by the plastic surgery team three months post WLE of the sebaceous carcinoma scar, and is currently doing well.

## Discussion

Several reports of invasive ductal carcinomas with sebaceous differentiation are found in the literature, however, reports of sebaceous carcinomas arising from the breast skin adnexa rather than the breast parenchyma are sparse. Svajdler et al. reported four cases of primary sebaceous carcinoma of the breast parenchyma; all showed at least 50% of sebaceous differentiation and no evidence of origin from the cutaneous adnexa [8]. Alzaraa et al. meanwhile, reported a case of sebaceous carcinoma arising from the skin of the breast, which clinically presented as an infected sebaceous cyst and was treated with excision, however, histopathological analysis revealed an incompletely excised sebaceous carcinoma [9]. In addition, the authors found that their 43-year-old patient had an underlying diagnosis of Muir-Torre syndrome, which will be further discussed below [9]. Cases of ocular and eyelid sebaceous carcinomas presenting as fungating lesions have been previously described [10,11]. In both cases, there has been a delay in presentation of six months and three years, respectively, which is similar to our case described above [10,11]. Extraocular presentations of sebaceous carcinomas as fungating tumours are rare but have been described before, and they present on the back and chest wall [12,13].

Sebaceous carcinomas also present diagnostic challenges for histopathologists. With standard H&E staining, sebaceous carcinomas can have similar appearances to other skin cancers such as BCCs and SCCs [4]. Distinctive features on H&E staining include undifferentiated basaloid cells with scalloped nuclei and multivacuolated clear cytoplasm [14]. BCCs can show focal sebaceous differentiation, but they also show peripheral palisading and artificial clefting around the basaloid islands [14]. Immunohistochemical staining can help distinguish sebaceous carcinomas from other benign and malignant skin lesions. They stain strongly positive for the androgen receptor, EMA, cytokeratin-7, and in 25% of cases, BER-Ep4; additionally, they tend to have higher expression of p53 and Ki-67 [4,14,15]. Histological diagnosis of sebaceous carcinomas is also aided by adipophilin immunohistochemical staining [16]. Expression of adipophilin is found in other solid tumours such as xanthelasmas, xanthogranulomata, and metastatic renal cell carcinomas; however, a membranous vesicular pattern of expression is more characteristic of sebaceous tumours [16].

Cutaneous sebaceous carcinomas can be associated with Muir-Torre syndrome. Muir-Torre syndrome, a variant of Lynch type 2 hereditary nonpolyposis colorectal cancer (HNPCC), exists where germline mutations in genes coding for mismatch repair (MMR) proteins are present, particularly MSH2 gene, predisposing patients to multiple other malignancies including colorectal and breast cancers [17,18]. Somatic mutations in proteins encoding for proteins in the MMR pathway may also develop. MMR protein mutations result in microsatellite instability, that leads to carcinogenesis, including the development of sebaceous carcinomas [19]. Other protein mutations can also underlie the pathogenesis of sebaceous carcinomas including transcription factors ZNF750, TP53, and Rb1 [19,20]. The diagnostic criteria for MTS are summarised in Table 1 [21]. Genetic testing for mutations in the MMR pathway can be considered and regular screening examinations should be offered to families with proven germline mutations [22]. In those proven not to have inherited the germline mutation, surveillance is not necessary [22].

**Table 1:** Diagnostic criteria for Muir-Torre syndrome

At least one of:	And:	Or all of the following:
Sebaceous carcinoma	1 or more visceral malignancies	Family history of Muir-Torre syndrome
Sebaceous epithelioma		Multiple visceral malignancies
Sebaceous adenoma		Multiple keratoacanthomas
Keratoacanthoma with sebaceous differentiation		

When it comes to excision, traditionally 5-6 mm excision margins have been advised for cutaneous sebaceous carcinomas, with a known high potential for recurrence [23]. However, even when this guidance is followed, local recurrence is reported to be 29%, with a 21% rate of distant metastases [24]. In our case, the skin MDT recommended a 2 cm WLE for the patient, which was relatively straightforward to achieve given the amount of skin laxity available in the breast.

## Conclusions

In conclusion, sebaceous carcinoma is a rare malignancy with most cases presenting in the head and neck region. Due to a high recurrence rate and high metastatic potential, it is important to manage these lesions appropriately, whilst at the same time looking for other synchronous malignancies and considering genetic testing. Our report describes the case of an 86-year-old lady presenting with a large fungating cutaneous sebaceous carcinoma affecting the skin of the left breast with two associated primary malignancies of the breast parenchyma. We present this report to demonstrate a unique presentation of an extraocular sebaceous carcinoma whilst at the same time raising awareness of the issues discussed above.
